# Prognostic Role of the Red Blood Cell Distribution Width (RDW) in Hodgkin Lymphoma

**DOI:** 10.3390/cancers12113262

**Published:** 2020-11-04

**Authors:** Ines Herraez, Leyre Bento, Raquel Del Campo, Adriana Sas, Rafael Ramos, Javier Ibarra, Francesc Mestre, Regina Alemany, Joan Bargay, Antonia Sampol, Antonio Gutierrez

**Affiliations:** 1 Department of Hematology, Son Llatzer University Hospital, 07198 Palma de Mallorca, Spain; ines.herraez@hsll.es (I.H.); rcampo@hsll.es (R.D.C.); jbargay@hsll.es (J.B.); 2 Health Research Institute of the Balearic Islands (IdISBa-IUNICS), 07120 Palma de Mallorca, Spain; 3 Department of Hematology, Son Espases University Hospital, 07120 Palma de Mallorca, Spain; leyre.bento@ssib.es (L.B.); adrianasas95@gmail.com (A.S.); antonia.sampolm@ssib.es (A.S.); 4 Department of Pathology, Son Espases University Hospital, 07120 Palma de Mallorca, Spain; rafaelf.ramos@ssib.es; 5 Department of Pathology, Son Llatzer University Hospital, 07198 Palma de Mallorca, Spain; jibarra@hsll.es; 6 Department of Radiotherapy, Son Espases University Hospital, 07120 Palma de Mallorca, Spain; franciscoj.mestre@ssib.es; 7 Department of Biology, University of Balearic Islands, 07122 Palma de Mallorca, Spain; regina.alemany@uib.es

**Keywords:** Hodgkin lymphoma, RDW, risk factors, survival, secondary malignancies

## Abstract

**Simple Summary:**

The red blood cell distribution width (RDW) increases in inflammatory conditions and is described as having a prognostic role in different types of cancer. As Hodgkin lymphoma (HL) has a proinflammatory background, we aim to study the prognostic role of RDW in HL. We report in a large retrospective series of homogenously treated HL, for the first time, that RDW is a simple, cheap, and easily available prognostic factor in HL, that identifies a group with worse EFS, OS, and a higher potential incidence of secondary malignancies. RDW seems to be related to most adverse prognostic factors in HL and this may make RDW a good candidate to be included in current or new prognostic scores for HL.

**Abstract:**

The red blood cell distribution width (RDW) is a parameter available from an automated blood count, which measures the degree of heterogeneity of erythrocyte volume and increases in inflammatory conditions. The prognostic role of RDW has been described in different types of cancers. Hodgkin lymphoma (HL) is a hematological malignancy, known to have a proinflammatory background. We aim to study the prognostic role of RDW in HL. We retrospectively analyzed 264 patients with HL from two hospitals in the Balearic Islands between 1990 and 2018. Higher levels of RDW were independently related to anemia, B-symptoms, and low albumin. In age ≥45 years, the presence of lymphopenia and higher RDW were independently associated with worse event-free survival (EFS) and overall survival (OS). Long-term incidence of secondary malignancies was significantly higher in patients with higher RDW, particularly lung cancer. In conclusion, we report for the first time that RDW is a simple, cheap, and easily available prognostic factor in HL that identifies a group with worse EFS, OS, and a higher potential incidence of secondary malignancies. RDW seems to be related to most adverse prognostic factors in HL, making RDW an excellent candidate to be included in prognostic scores for HL.

## 1. Introduction

Hodgkin lymphoma (HL) is a hematological malignancy characterized by few neoplastic cells called Reed-Sternberg inside an inflammatory microenvironment [1]. Standard therapy regimens cure approximately 80% of patients, but the other 20% will require salvage therapy [2]. Identifying factors that could improve the early detection of these refractory patients is very important to improve risk stratification and individualize treatment [3].

The red blood cell distribution width (RDW) is a simple blood test parameter that reflects the size diversity of red blood cells (anisocytosis) in peripheral blood and traditionally was used to study anemias [4]. In the last decade, higher levels of this parameter have been described as an adverse prognostic factor in cardiovascular diseases, inflammation, and cancer [5,6,7,8,9,10,11].

In cancer patients, higher values of RDW could be associated with a higher degree of inflammation. Increased levels of cytokines may modify iron metabolism by increasing levels of hepcidin and oxidative stress. Simultaneously, erythropoietin production is reduced, resulting in more anisocytosis and higher values of RDW [12,13].

Notably, RDW has been reported to have a prognostic role in several lymphoproliferative diseases, such as chronic lymphocytic leukemia (CLL), diffuse large B-cell lymphoma (DLBCL), mantle cell lymphoma (MCL), or multiple myeloma [5,14,15,16,17,18,19,20,21,22]. Several groups recently proposed the inclusion of RDW in prognostic scores in DLBCL [23,24].

However, there are scarce data for HL, where a proinflammatory background is a key event in pathogenesis and physiopathology [25]. We aim to analyze the potential prognostic role of RDW in HL.

## 2. Results

### 2.1. Patient Characteristics

A total number of 264 patients with classic HL homogeneously treated with ABVD +/− RT were retrospectively analyzed at the time of diagnosis, at Son Espases (*n* = 165) and Son Llatzer (*n* = 99) University Hospitals in the Balearic Islands, between 1990 and 2018. The presenting features of the patients are shown in Table 1. The median age was 37 years (14–83 years), 52% of patients had an advanced stage, 16% had bulky disease, 28% had an Eastern Cooperative Oncology Group Performance Status (ECOG PS) >1, and 19% had an IPS >3. All prognostic factors of IPS are also shown in Table 1.

### 2.2. Analysis of the Prognostic Role of RDW

**Figure 1:**
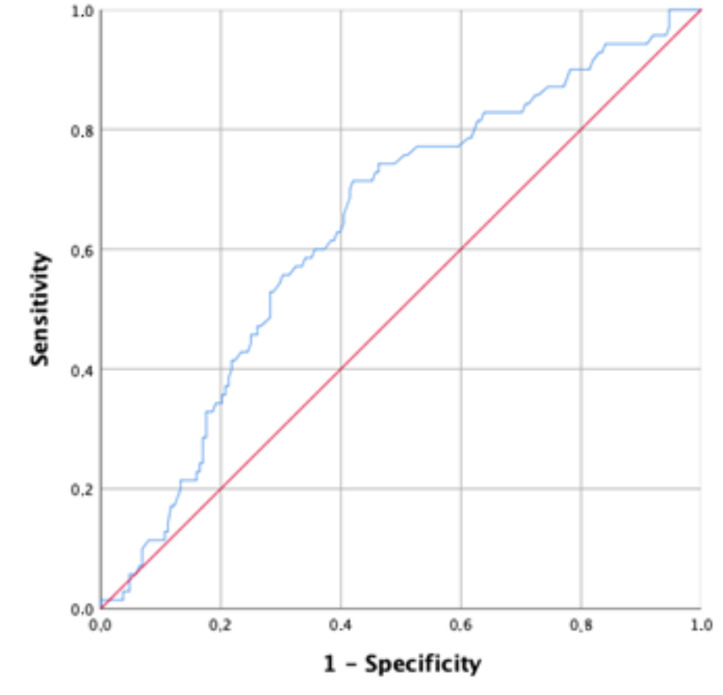
ROC analysis about the prognostic role of RDW on progression or death of any cause.

As shown in Table 1, the median RDW in the cohort was 13.9 (range, 10.6–23.9). To evaluate the ability of RDW in predicting a worse outcome, we considered the progression or death of any cause. As normal values changed with the center and the time, we standardized the values as a ratio of the upper normal value in each center and time. Using a ROC analysis, we obtained an optimal cutoff of 0.95 of sRDW with an area under the curve of 0.64 (CI95%: 0.57–0.71) (*p* = 0.001) (Figure 1).

### 2.3. RDW and Main Prognostic Factors in HL

Additionally, we studied the relationship between RDW and the main prognostic factors in HL. We found that patients with sRDW >0.95 were significantly older patients, with more advanced disease, with a higher incidence of B-symptoms, with worse ECOG PS, and several worse adverse prognostic factors such as higher erythrocyte sedimentation rate (ESR), lower albumin levels, lower counts of lymphocytes and low hemoglobin (Hb) levels at diagnosis. Multivariate analysis showed that sRDW >0.95 was independently associated to patients with anemia (Hb < 10.5) (RR 5.9; CI95%: 2–16.9; *p* = 0.001), B-symptoms (RR 2.5; CI95%: 1.3–4.9; *p* = 0.007) and low albumin level (RR 2.2; CI95%: 1.1–4.4; *p* = 0.019) (Table 2).

### 2.4. Response and Survival Analysis

With frontline therapy, 88% of the patients reached complete response (CR), 4% had a partial response (PR), and 8% a stable/progressive disease (SD/PD). With a median follow-up of 81 months (range, 11–352), six-year event-free survival (EFS) and overall survival (OS) were 74% (CI95%: 72–77) and 86% (CI95%: 84–88), respectively.

**Figure 2:**
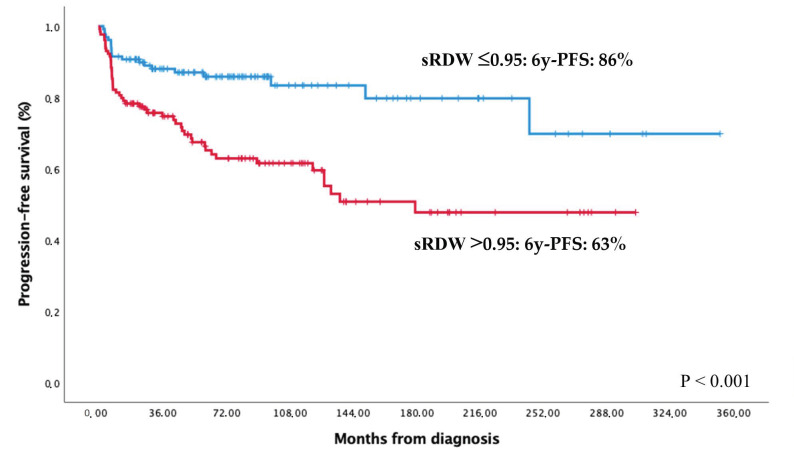
Progression-free and overall survival according to sRDW.

Table 3 shows the univariate and multivariate survival analysis in which we included all variables found to be significant in the univariate analysis and sex as a potential confounding factor. Briefly, EFS was significantly influenced by age, AA stage, B-symptoms, ECOG PS, IPS, and all related prognostic factors, excluding sex. Among the alternative inflammatory biomarkers tested: RDW (Figure 2), ESR, and RCP were also related to EFS. However, only age ≥ 45 years, sRDW > 0.95, and the presence of lymphopenia were independently associated with a worse EFS. CR tended to be higher in patients with sRDW ≤ 0.95: 92% vs. 84% (*p* = 0.051).

In the case of OS, univariate analysis showed the influence of age, B-symptoms, ECOG PS, IPS, and all related factors, excluding sex and leucocytes. Regarding alternative inflammatory biomarkers, only CRP and sRDW seemed to significantly influence OS. Multivariate analysis found the same three prognostic factors (age ≥ 45 years, sRDW > 0.95, and lymphopenia) independently associated with a worse OS, together with the male sex, that was included in the analysis as a potential confounding variable (Table 3).

Furthermore, the long-term incidence of second malignancies was significantly higher in patients with sRDW > 0.95: 15 (12%) vs. 4 (3%) (*p* = 0.015). Particularly, the incidence of lung cancer was much higher (5% vs. 1%) in patients with sRDW > 0.95 (Table 4). Unfortunately, the low incidence of specific secondary malignancies does not allow us to draw more definitive conclusions regarding the specific role of sRDW or other factors in the incidence of each type of malignancy. While anecdotal, all three patients with head and neck malignancies were previously treated with radiotherapy (2 with sRDW ≤ 0.95).

Using binary logistic regression, we studied the prognostic factors related to the incidence of secondary malignancies. In the univariate analysis we observed a significantly higher risk of secondary malignancies in older patients (*p* = 0.013), with Hb < 10.5 g/dL (*p* = 0.013), and sRDW > 0.95 (*p* = 0.014), but not radiotherapy administration (*p* = 0.15). However, we performed a multivariate analysis including all significant prognostic factors from the univariate analysis as well as radiotherapy as a potential confounding factor, and we found that only older age (RR: 1.03; *p* = 0.018), sRDW > 0.95 (RR: 3.84; *p* = 0.047) and radiotherapy administration (RR: 3.81; *p* = 0.014) were independently related to a higher incidence of secondary malignancies.

## 3. Discussion

To our knowledge, this is the first published report about the main prognostic role of RDW in HL. We previously presented part of this data at the 58th Annual Meeting of the American Society of Hematology [25]. In our study, RDW strongly correlated with main prognostic factors in HL, and sRDW > 0.95 is shown to be an independent adverse prognostic factor for EFS and OS.

RDW is an automatically measured index of the heterogeneity of the erythrocytes [4]. Traditionally, this parameter was used for the differential diagnosis of anemias [26]. In cancer, anemia could be present due to inflammation, and after treatment [27].

Physiological conditions that could increase the RDW levels include aging, erythropoietin, pregnancy, black ethnicity, and physical exercise [13]. In previous years, an increase in RDW levels was described as an adverse prognostic factor that increases mortality in the general population, associated with many acute and chronic conditions, in which inflammation represents a critical factor, including metabolic, cardiovascular, and thrombotic disorders [13].

RDW, in cancer, reflects chronic inflammation and poor nutritional status [28]. Certain studies support that cytokines play a central role in RDW, being associated with advanced stages and higher mortality. It has been related to different inflammatory markers such as interleukin-6, ESR, CRP, soluble tumor necrosis factor receptors I and II, and soluble transferrin receptor [12]. Elevated levels of proinflammatory cytokines led to inadequate production of erythropoietin, impaired erythrocyte maturation, a poor nutritional status (hypoalbuminemia), and increased levels of hepcidin and oxidative stress. These are different biological mechanisms that may lead to higher values of RDW [28].

RDW has been shown to confer a worse prognosis in many types of hematological malignancies, including lymphoproliferative and myeloproliferative disorders. In aggressive lymphomas, such as DLBCL, elevated levels of RDW at diagnosis were associated with worse ECOG PS, B-symptoms, and higher IPI. They predicted a poorer prognosis [15,22]. It has been described as an independent adverse prognostic factor at diagnosis in MCL, and RDW also improved the prognostic stratification based on the simplified Mantle Cell International Prognostic Index (sMIPI) [16]. In extranodal NK/T nasal-type lymphoma, high RDW at diagnosis has been associated with poorer clinical outcomes in patients treated with radiotherapy-based schedules [29].

In indolent lymphoproliferative disorders, such as multiple myeloma, RDW at diagnosis has been described to predict the outcome and correlate with response to therapy [17,18,19,21]. Furthermore, in CLL, elevated RDW levels at diagnosis were associated with adverse prognostic factors such as advanced disease [14]. In hairy cell leukemia, high RDW was associated with active disease both at diagnosis and after receiving treatment [30].

Regarding myeloid malignancies, a prognostic role at diagnosis has been described in chronic myeloid leukemia as a biomarker for risk stratification and its ability to predict response to treatment [31]. In myelodysplastic syndromes, high RDW values in patients with less than 5% blasts at diagnosis, was an independent prognostic factor. Approximately 30% of patients classified as IPSS-R lower-risk showed similar outcomes to those with higher-risk IPSS-R [32,33].

Most variables independently influencing PFS and OS detect HL’s immune relationships (sRDW > 0.95, lymphopenia and older age). RDW is more related to systemic inflammation, while lymphopenia may be more associated with immune dysfunction. Older age may be related to both situations.

In our series, we found a strong relationship between RDW and most prognostic factors in HL. Multivariate analysis showed that a higher RDW was independently associated with anemia, higher CPR, and low albumin. However, the most important characteristic of RDW is that it is a cheap and easily available prognostic factor that may be obtained from automatic blood cell counts at the time of diagnosis.

More importantly, RDW was independently associated with EFS and OS. We also found a relationship with the long-term development of secondary malignancies, a critical adverse prognostic factor in a malignancy such as HL with good long-term survival. RDW is increased in patients with a higher proinflammatory background, more prone to adverse events, and shorter PFS as well as with a higher incidence of cancer in general.

Some of the present study’s limitations include a retrospective analysis performed in two different centers over almost 30 years. However, we tried to minimize these limitations by using a significant sample (*n* = 264) without selection bias, homogenously treated (ABVD), and by standardizing the RDW values.

## 4. Materials and Methods

### 4.1. Patients and Sample Selection

We selected patients with classic HL homogeneously treated with ABVD (adriamycin, bleomycin, vinblastine, and dacarbazine) +/− radiotherapy (RT) at Son Espases and Son Llatzer University Hospitals in the Balearic Islands, between 1990 and 2018. To avoid selection bias, we selected patients from the databases of the Services of Pathology and Pharmacy. Those patients treated with different schemes were excluded. This study was approved by the Ethics Committee of the Balearic Islands with the number IB4071/19.

### 4.2. Clinical and Laboratory Prognostiz Factors

Clinical variables were obtained from medical records including main prognostic factors in main prognostic indexes in HL: age, gender, Ann Arbor Stage, lactate dehydrogenase (LDH) and β-2 microglobulin (B2M) serum levels, extranodal sites, B-symptoms, Eastern Cooperative Oncology Group performance status (ECOG PS), bulky disease and main variables of automated blood counts. As RDW values have been obtained using different techniques and measurement systems in different centers and periods, we standardized them using the normal reference values of each determination, generating a standardized RDW (sRDW).

Main prognostic scores, International Prognostic Score (IPS), and those from the European Organization for Research and Treatment of Cancer (EORTC) and the German Hodgkin Lymphoma Study Group (GHSG) were calculated. Response assessment was done using Cheson [34] or Lugano criteria [35] in the corresponding time periods.

### 4.3. Statistical Methods

Qualitative or binomial variables were expressed as frequencies and percentages. Comparisons between qualitative variables were made using the Fisher Exact Test or Chi-square. Comparisons between quantitative and qualitative variables were performed through non-parametric tests (U of Mann-Whitney or Kruskal-Wallis). Receiver operating curve (ROC) analysis was used to obtain and optimal sRDW cutoff for progression or death of any cause. The binary logistic regression was used to find out the risk factors associated with sRDW and those associated with a higher risk of secondary malignancies.

Time to event variables were estimated according to the Kaplan-Meier method, and the log-rank test performed comparisons between variables of interest. Multivariate analysis with the variables that were significant in the univariate analysis and potential confounding factors was carried out according to the Cox proportional hazard regression model. A backward stepwise Cox multivariate analysis was performed to determine factors independently associated with PFS and OS. All p values reported were 2-sided, and statistical significance was defined at *p* < 0.05. Statistical analysis was performed using a statistical package program (SPSS Inc, Chicago, IL, USA).

## 5. Conclusions

In conclusion, we report for the first time that RDW is a simple, cheap, and easily available prognostic factor in HL, that identifies a group with worse EFS, OS, and a higher potential incidence of secondary malignancies. RDW seems to be related to most adverse prognostic factors in HL, making RDW an excellent candidate to be included in prognostic scores for HL. We are currently exploring new prognostic scores in HL including variables easy to obtain from the automated blood counts, such as RDW.

## Figures and Tables

**Table 1:** Patients characteristics.

Characteristics	N (%)	Missing Data
Median age (range)	37 (14–83)	0 (0%)
Age ≥ 45: Age > 60:	87 (32%) 34 (13%)	0 (0%)
Sex: M/F	156 (59%)/108 (41%)	0 (0%)
Diagnosis: - NS - MC - LR - LD - Other - Unknown	183 (69%) 45 (17%) 13 (5%) 7 (3%) 6 (2%) 10 (4%)	10 (4%)
Ann Arbor Stage: - I–II - III–IV	126 (48%) 138 (52%)	0 (0%)
B-symptoms:	115 (44%)	0 (0%)
Bulky disease:	42 (16%)	1 (0%)
ECOG PS: - 0–1 - >1	190 (72%) 74 (28%)	0 (0%)
ESR: - Normal - High	99 (40%) 147 (60%)	18 (7%)
Albumin: - 40 or more - <40	117 (49%) 120 (51%)	27 (10%)
Leucocytes ≥ 15,000/µL	35 (13%)	0 (0%)
Lymphocytes < 600/µLor < 8% of total leucocytes	45 (17%)	0 (0%)
Hb < 10.5 g/dL	61 (23%)	1 (0%)
Median RDW (range)	13.9 (10.6–23.9)	6 (2%)
GHSG > 0:	202 (79%)	10 (4%)
EORTC > 0:	186 (73%)	10 (4%)
IPS: - 0–3 - >3	214 (81%) 49 (19%)	1 (0%)

NS: nodular sclerosing; MC: mixed cellularity; LR: lymphocyte rich; LD: lymphocyte depleted; ECOG PS: Eastern Cooperative Oncology Group Performance Status; ESR: erythrocyte sedimentation rate; Hb: hemoglobin; RDW: red blood cell distribution width; GHSG: German Hodgkin Study Group; EORTC: European Organization for Research and Treatment of Cancer; IPS: international prognostic score.

**Table 2:** Analysis of the relationship of RDW and main prognostic factors in HL.

Characteristics	sRDW 0–0.95	sRDW > 0.95	*P*
Median age (range)	33 (14–83)	40 (15–83)	0.014
Sex: M/F	70 (54%)/59 (46%)	82 (64%)/47 (36%)	0.16
Ann Arbor Stage III–IV	42 (33%)	93 (72%)	<0.001
B-symptoms:	30 (23%)	83 (64%)	<0.001
Bulky disease:	19 (15%)	21 (16%)	0.86
ECOG PS > 1	24 (19%)	49 (38%)	0.001
Elevated ESR:	45 (39%)	100 (80%)	<0.001
Albumin < 40:	33 (29%)	86 (72%)	<0.001
Leucocytes ≥ 15,000/µL	12 (9%)	22 (17%)	0.097
Lymphocytes < 600/µL or < 8% of total leucocytes	7 (5%)	37 (29%)	<0.001
Hb < 10.5 g/dL	6 (5%)	55 (43%)	<0.001
IPS > 3	1 (1%)	47 (37%)	<0.001

sRDW: standardized red blood cell distribution width; M/F: male/female; ECOG PS: Eastern Cooperative Oncology Group Performance Status; ESR: erythrocyte sedimentation rate; Hb: hemoglobin; IPS: international prognostic score.

**Table 3:** Univariate and multivariate survival analysis for EFS and OS.

Univariate Analysis				
Factor	6-year EFS (95% CI)	*p*	6-year OS (95% CI)	*P*
Age: - 14–44 - ≥45	80% (74–86) 63% (51–74)	0.005	93% (90–97) 70% (59–81)	<0.001
Age: - 14–60 - >60	78% (72–83) 46% (46–11)	0.002	90% (86–94) 54% (32–76)	<0.001
Sex: - Male - Female	75% (68–82) 74% (65–83)	0.33	85% (79–91) 87% (80–95)	0.1
Ann Arbor stage: - I–II - III–IV	83% (76–90) 67% (58–75)	0.002	91% (85–96) 81% (74–89)	0.1
B symptoms: - No - Yes	82% (75–88) 65% (55–75)	0.001	89% (83–94) 82% (74–90)	0.031
ECOG PS: - 0–1 - 2–4	78% (72–84) 65% (51–79)	0.002	88% (83–92) 76% (62–90)	0.007
IPS: (All stages) - 0–3 - >3	78% (72–84) 58% (43–74)	0.002	88% (83–92) 77% (64–91)	0.008
IPS: (Advanced HL) - 0–3 - >3	68% (58–79) 62% (47–78)	0.26	83% (74–92) 77% (63–92)	0.072
ESR: - Normal - High	82% (75–90) 68% (60–77)	0.003	90% (84–96) 83% (76–90)	0.079
CRP: - Normal - High	89% (79–99) 68% (60–77)	0.005	95% (87–100) 83% (76–90)	0.047
sRDW: - ≤0.95 - >0.95	86% (80–92) 63% (54–72)	<0.001	94% (90–98) 78% (70–86)	0.001
B2M: - Normal - High	80% (73–87) 63% (52–75)	0.003	94% (90–98) 74% (63–84)	<0.001
Albumin: - ≥40 - <40	84% (77–91) 66% (57–76)	0.001	93% (87–98) 83% (76–91)	0.02
Leucocytes: - <15000 - ≥15000	77% (71–83) 58% (41–75)	0.006	87% (82–92) 80% (66–93)	0.13
Lymphocytes: - >600/8% - <600/8%	80% (74–85) 48% (32–65)	<0.001	89% (88–95) 68% (52–85)	<0.001
Hb: - ≥10.5 - <10.5	81% (75–87) 51% (37–66)	<0.001	88% (83–93) 79% (68–91)	0.044
**Multivariate Analysis**				
	**PFS**		**OS**	
Factor	HR (95% CI)	p	HR (95% CI)	P
Lymphocytes < 600/8%	2.2 (1.3–4)	0.006	2.7 (1.2–5.7)	0.013
RDW > 0.95	2.3 (1.2–4.3)	0.007	3 (1.1–8.1)	0.027
Age ≥ 45	1.8 (1.1–3)	0.022	5.7 (2.6–12.5)	<0.001
Male sex	---	---	2.5 (1–6.1)	0.049

EFS: event-free survival; OS: overall survival; ECOG PS: Eastern Cooperative Oncology Group Performance Status; IPS: international prognostic score; ESR: erythrocyte sedimentation rate; CRP: C reactive protein; sRDW: standardized red blood cell distribution width; B2M: B2microglobulin; Hb: hemoglobin.

**Table 4:** RDW and incidence of second malignancies.

	sRDW ≤ 0.95	sRDW > 0.95	*p*
Second malignancies	4 (3.1%)	15 (11.6%)	0.015
Lung cancer Head and neck Other	1 (0.8%) 2 (1.6%) 1 (0.8%)	7 (5.4%) 1 (0.8%) 7 (5.4%)	0.02

sRDW: standardized red blood cell distribution width.
